# Supplementary figures and images for: Study of Humoral Responses against Lomentospora/Scedosporium spp. and Aspergillus fumigatus to Identify L. prolificans Antigens of Interest for Diagnosis and Treatment
Source: Vaccines (Basel). 2019 Dec 10;7(4):212. doi: 10.3390/vaccines7040212 (PMC6963885; doi:10.3390/vaccines7040212)

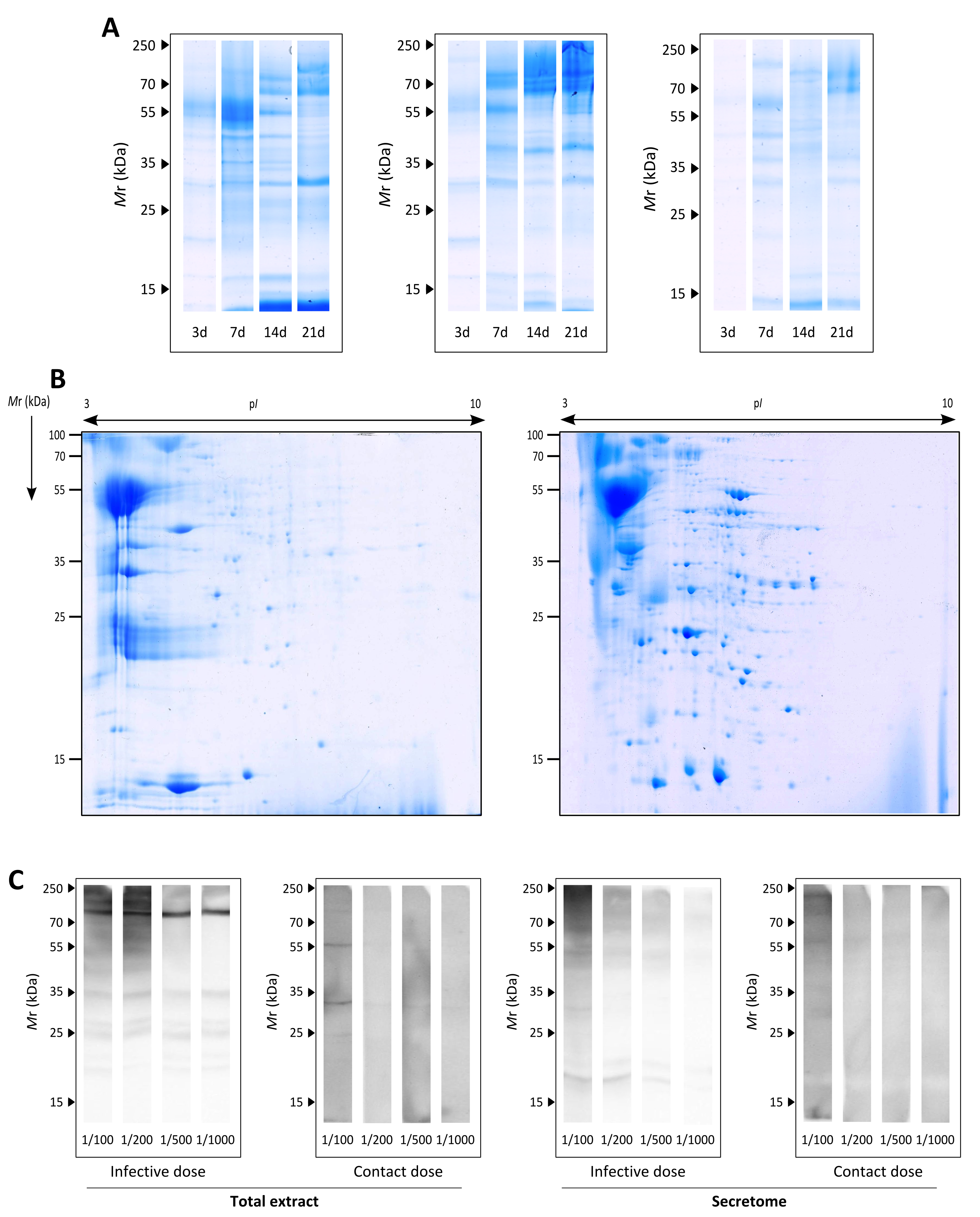

Supplement: Supplementary file 1 [file vaccines-07-00212-s001.zip › vaccines-643017-supplementary/Figure S1.png]

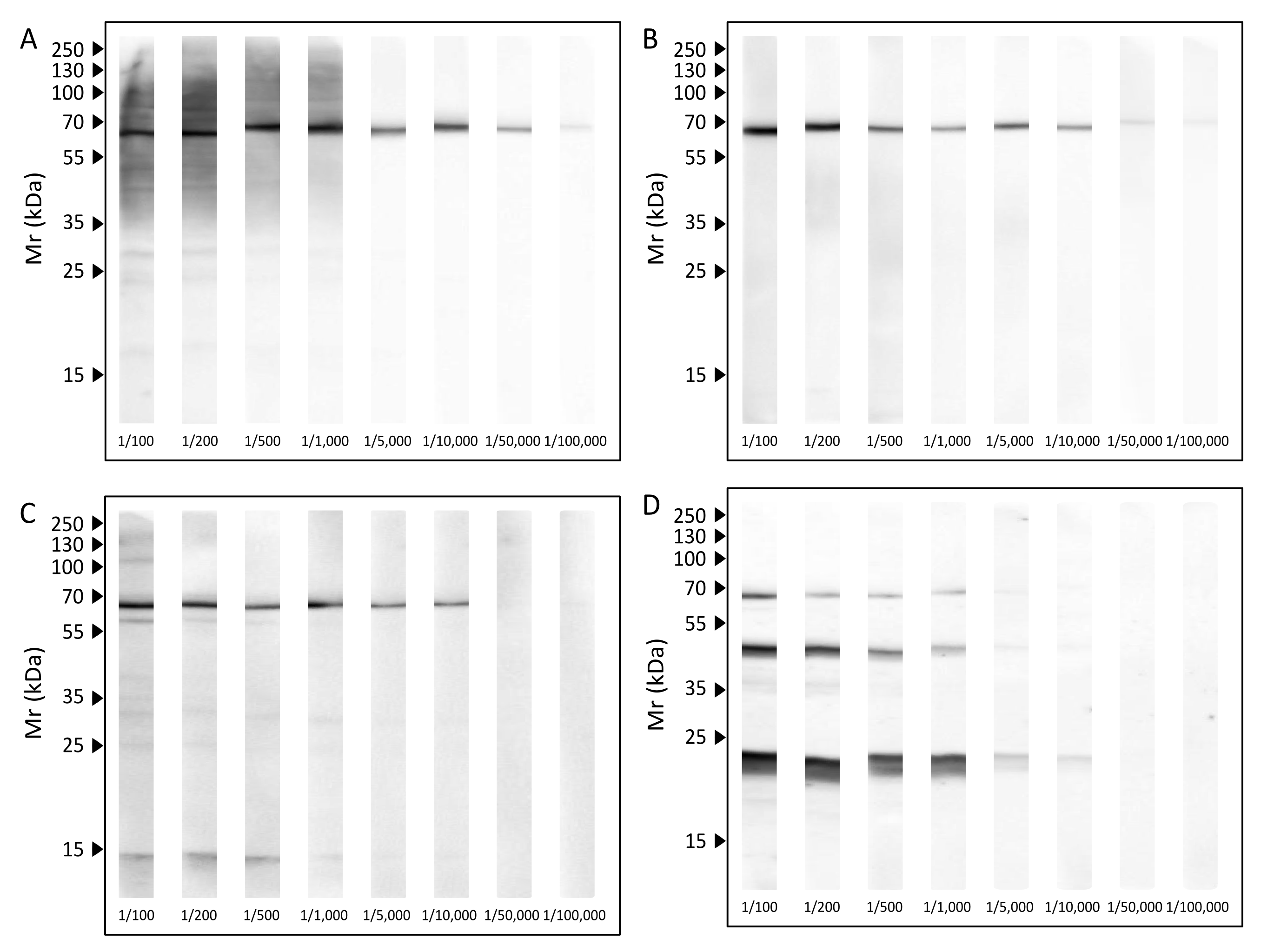

Supplement: Supplementary file 1 [file vaccines-07-00212-s001.zip › vaccines-643017-supplementary/Figure S2.png]
